# Evaluation of QUASAR Insight Phantom for daily imaging QA of MRgRT linacs

**DOI:** 10.1002/acm2.70463

**Published:** 2026-02-16

**Authors:** Eli Grant, Jiayi Liu, Ronak Etemadpour, Chengyu Shi, Huisi Ai, Percy Lee, Terence Williams, An Liu, Borna Maraghechi

**Affiliations:** ^1^ Department of Radiation Oncology City of Hope National Medical Center Irvine California USA; ^2^ Department of Radiation Oncology University of California Irvine Medical Center Orange California USA; ^3^ Department of Radiation Oncology City of Hope National Medical Center Duarte California USA

**Keywords:** adaptive radiation therapy, linac, magnetic resonance‐guided radiation therapy, MR imaging, phantom, quality assurance

## Abstract

**Purpose:**

Magnetic resonance imaging (MRI) and its importance in modern radiation therapy requires a standardized daily quality assurance (QA) procedure that is both comprehensive and efficient. Current imaging QA guidelines require a more inclusive analysis of imaging characteristics, as well as a simplification of setup procedures. We have studied the feasibility of using the Insight Phantom to provide consistent images for analyses of uniformity, slice thickness, spatial resolution, and distortion across a large field of view (FOV).

**Methods:**

The large imaging region of the Insight Phantom was imaged using 3D True Fast Imaging with Steady State Precession (TruFi) and analyzed using the Modus QA software. The base and upright section were imaged in coronal, axial, and sagittal orientations over 3 months.

**Results:**

Geometric distortion analysis yielded average maximum distortions within a 210 mm diameter of 0.57, 0.73, and 0.45 mm for axial, sagittal, and coronal orientations, respectively. Similarly, within a 310 mm diameter, average maximum distortions were 0.58, 0.73, and 0.74 mm with no distortions greater than or equal to 1 mm. Average modulation transfer function (MTF) values were 0.431 ± 0.007, 0.436 ± 0.007, and 0.433 ± 0.010 lp/mm for axial, sagittal, and coronal images, respectively. Finally, slice thickness values were consistent at 2.19 ± 0.04, 2.22 ± 0.06, and 2.24 ± 0.07 mm for axial, sagittal, and coronal imaging orientations, respectively. Images were repeatable and setup procedures were quick and straightforward, enhancing therapist workflow and efficiency.

**Conclusion:**

The Insight Phantom's large coverage allows a more in‐depth analysis of the MRI's large FOV, and its simple setup ensures a repeatable daily QA procedure for therapists. The software also provides a comprehensive analysis of key imaging parameters in a detailed report that can be used to monitor MRI functionality and quality.

## 1 INTRODUCTION

The integration of magnetic resonance imaging (MRI) technology with linear accelerators (linacs) offers online adaptive radiation therapy (ART), marking a significant leap in the precision treatment of cancer. The MR‐Linac not only enhances tumor targeting accuracy across various cancer sites by accommodating daily anatomical changes but also improves patient outcomes through real‐time motion management.^1^, ^2^, ^3^

The critical role of imaging in magnetic resonance‐guided radiation therapy (MRgRT) highlights the importance of rigorous quality assurance (QA) to ensure reliability and accurate treatments. As MRgRT relies heavily on MRI for both planning and guiding radiation treatment, any inaccuracies in MRI can lead to significant discrepancies in dose delivery.^4^, ^5^, ^6^, ^7^, ^8^, ^9^ Comprehensive QA procedures are therefore important for verifying the performance and accuracy of MRI systems used in radiation oncology, ensuring that they meet the requirements necessary for precise and effective treatment.

A major challenge in integrating MRI with radiation therapy is the presence of geometric distortions in MRI images. These distortions arise from system‐related factors such as field inhomogeneities and gradient nonlinearities, as well as patient‐specific factors like magnetic susceptibility differences among tissues.^10^, ^11^ To address these issues, robust methods for assessing and correcting geometric distortions across the entire bore are crucial. This includes the development of phantoms and algorithms that can accurately measure and track distortions, thereby preserving the spatial integrity of MRI‐based treatment planning and delivery.

In response to the need for standardized QA practices in MRgRT, guidelines and reports have been developed. The American College of Radiology (ACR) provides comprehensive guidelines for MRI imaging and offers an MRI accreditation program that sets quality standards for MRI facilities. AAPM Task Group 284 (TG‐284) discusses the integration and optimization of MRI simulation within the field of radiotherapy. Here, ACR is referenced in the context of MR safety, as illustrated with established guidelines and standards for MR imaging in a radiotherapy setting.^12^

Among the tools used for MRI QA, the ACR MRI phantom is widely recognized for its role in imaging QA processes.^13^, ^14^ Designed to evaluate the performance of MRI systems, the ACR MRI phantom is a cylinder with a diameter of 203.2 mm and a length of 173.40 mm, which helps assess imaging characteristics, such as geometric accuracy, image uniformity, and spatial resolution. However, the ACR phantom's relatively small imaging size limits its ability to detect geometric inaccuracies across larger fields of view.

Another commonly used tool for MRI QA in radiation therapy is the Magphan RT phantom (The Phantom Laboratory, Salem, NY, USA). These phantom measures a variety of imaging characteristics including geometric distortion, uniformity, and slice thickness over the entire imaging volume of 35 × 39 × 21 cm^3^. Its usage in daily QA practices helps in identifying geometric inaccuracies and thus ensuring that MRgRT treatments are based on reliable images. Furthermore, Magphan's web‐based software called “Smari” allows for the fully automated and efficient analysis of collected QA testing data.^15^, ^16^, ^17^ Although the Magphan RT phantom provides a practical and time‐saving way to perform mandatory machine QA, limitations arise that pertain to the phantom's size and geometry. Loading the phantom onto the couch can be difficult due to its bulk and a weight of just under 80 pounds, which may require special equipment to carry it. This can also lead to delays caused by errors in positioning. When wrapped in RF coils, the phantom's oval shape can cause a slight tilt,^16^ leading to small rotational misalignments.

In this study, we report daily QA results acquired with the QUASAR Insight Phantom over 3 months on a ViewRay MRIdian MR‐Linac. Our analysis evaluates stability, day‐to‐day variability, and sequence‐specific artifacts, with the goal of determining the phantom's suitability for routine daily QA in MRgRT. By presenting this dataset, we aim to provide clinically relevant guidance on the phantom's performance and its role in supporting accurate, efficient, and practical MRgRT workflows.

The QUASAR Insight Phantom (Modus QA) emerges as another QA phantom with an imaging region that covers a large FOV. With a 426 mm radius upright section and a 430 × 510 mm base, the phantom's design allows for a comprehensive analysis of uniformity, slice thickness, spatial resolution, and distortions across a wide imaging area. This extensive coverage allows for the detection of geometric inaccuracies over a larger field of view for treatments that require a large field of view. Although geometric distortion measurements are not typically required in routine daily QA, the use of the Insight Phantom and associated software enabled us to assess distortion, in addition to uniformity, slice thickness, and spatial resolution, from a single acquisition. This integration allowed a more comprehensive imaging QA evaluation without increasing workload, thereby providing supplementary information beyond the minimum daily QA requirements.

While prior work by Lewis et al.^18^ has demonstrated the feasibility of using the Insight Phantom for imaging QA, these studies primarily focused on initial characterization or periodic evaluations rather than longitudinal clinical performance. In contrast, our study presents a 3‐month dataset obtained from routine daily QA, allowing for a detailed assessment of reproducibility and day‐to‐day stability under real clinical operating conditions.

This study utilizes the QUASAR Insight Phantom to evaluate its performance in daily QAimaging on a ViewRay MRIdian MR‐Linac. By analyzing and presenting the results, we aim to demonstrate the phantom's suitability for routine imaging QA, providing guidance for medical professionals and contributing to improved imaging quality, patient care, and workflow efficiency.

## 2 METHODS

### 2.1 Insight Phantom

**FIGURE 1:**
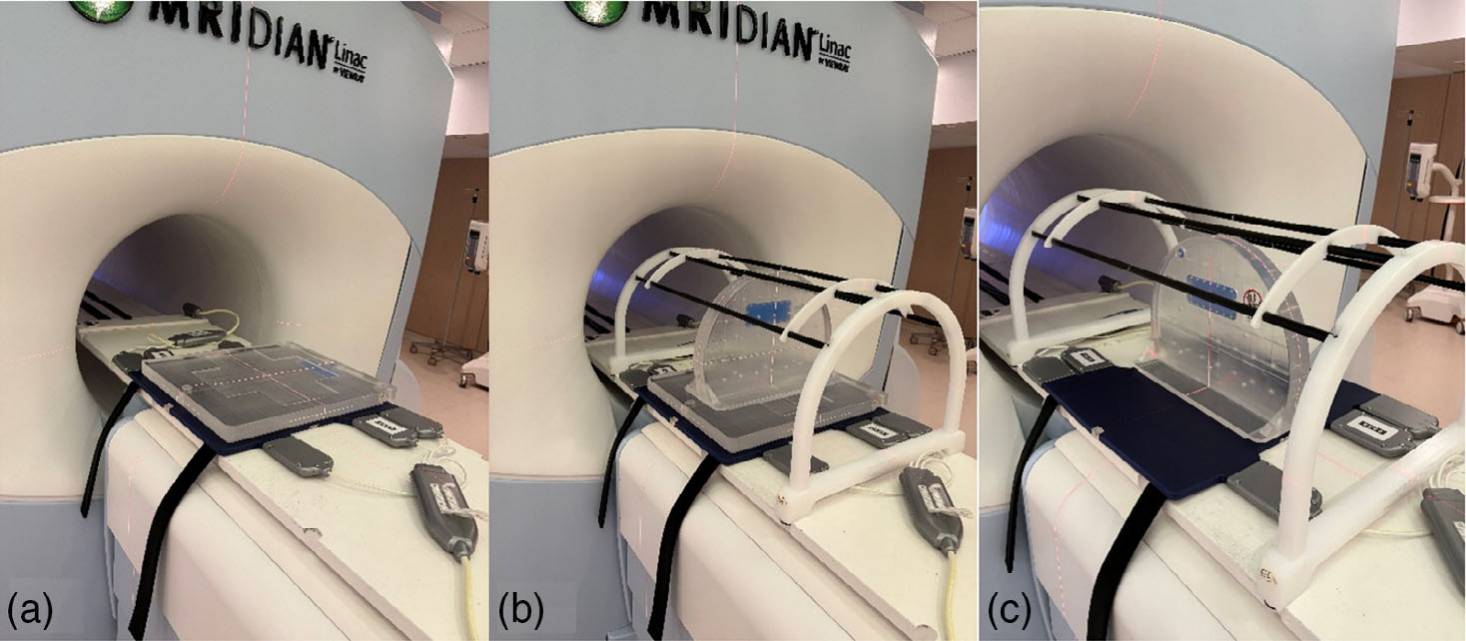
The Insight Phantom in the coronal (a), axial (b), and sagittal (c) orientations. Images (b) and (c) are shown with the Phantom Coil Bridge.

In this study, we utilized the QUASAR MRgRT Insight Phantom (Modus Medical Devices Inc. (Modus QA), London, Ontario, Canada). The phantom consists of an upright section and a base, as well as a phantom coil bridge. The upright section is a truncated circular slab with a radius of 426 mm and a thickness of 52 mm, held up by a base of 510 mm by 430 mm. The base itself is 41 mm thick. Both sections of the phantom include similar image quality structures, including ramps, spatial resolution test regions, a geometric distortion grid, a flood field, and slice position accuracy test zones. To hold the anterior RF array coil, a coil bridge accessory is provided as shown in Figure 1. The upright section is designed for axial and sagittal imaging orientations and the base for coronal imaging orientations.

The QUASAR MRgRT Insight Phantom software used in this study aids with the automated analysis of MR images of the Insight Phantom. For easy and quick interpretation, the results of the measurements for different imaging characteristics are highlighted in green, yellow, and red for passed, warning, and failed, respectively. In the case of the phantom being misaligned, the software also computes the lateral, longitudinal, and vertical offsets as well as any rotational errors.

### 2.2 Imaging setup

The Insight phantom was used in this work for daily imaging QA. All daily imaging QA acquisitions were performed on a 0.35 T ViewRay MRIdian MR‐Linac (ViewRay, Mountain View, California, USA) with TDS version A3i, using the clinical mode. Figure 1 shows the clinical daily imaging QA set up for both the base and upright section of the phantom. The base of the phantom was imaged in the coronal setup position, while the upright section was imaged in the sagittal and axial setup positions. For consistent and thorough data collection, the phantom orientation alternated between coronal, axial, and sagittal setup positions on a weekly basis. For all QA acquisitions, the gantry was parked at 0° to maintain consistency. For the analysis of long‐term trends in measured values, 50 scans were collected for each imaging orientation, totaling 150 scans over the course of 3 months.

For consistency and quality, imaging parameters were optimized to provide a more comprehensive representation of the entire phantom. The clinical TruFi scan was used for this study with scan parameters shown in Table 1 below.

**TABLE 1:** TruFi MRI clinical scan parameters used to scan the Insight Phantom.

ViewRay TruFi scan parameters	
TR (ms)	3.36
TE (ms)	1.43
Flip angle (°)	60
FOV (mm^3^)	500 × 500 × 500
Matrix	300 × 300 × 300
Resolution (mm^3^)	1.5 × 1.5 × 1.5
rBW (Hz/Px)	538
NSA	1
Acquisition time (s)	120

*Note*: TruFi MRI clinical scan parameters used to scan the Insight Phantom.

Abbreviations: FOV, field of view; MRI, magnetic resonance imaging; TruFi, 3D True Fast Imaging with Steady State Precession.

The field of view (FOV) was expanded to 500 mm × 500 mm × 500 mm, ensuring the inclusion of the entire phantom structure within the imaging scope. The voxel size was set to be 1.5 mm isotropic, providing detailed spatial resolution. The fastest scan speed was selected with a total scan time of around 120 s to increase efficiency while maintaining a high imaging quality.

### 2.3 Image analysis

#### 2.3.1 Phantom alignment

For phantom alignment, the software provides quantitative feedback on both the translational (position) and rotational (twist) displacement. Position refers to the distances from the center of the phantom (indicated by a crosshair in the images) relative to the slice axis. The distances from the slice axis to the center are given in millimeters (mm) along the *x*, *y*, and *z*‐axes, which correspond to the lateral, longitudinal, and vertical displacements, respectively. The twist refers to the rotational deviation of the phantom along all three axes. It is measured in degrees and represents to what extent the phantom has been twisted/rotated about the slice axis.

#### 2.3.2 Uniformity

**FIGURE 2:**
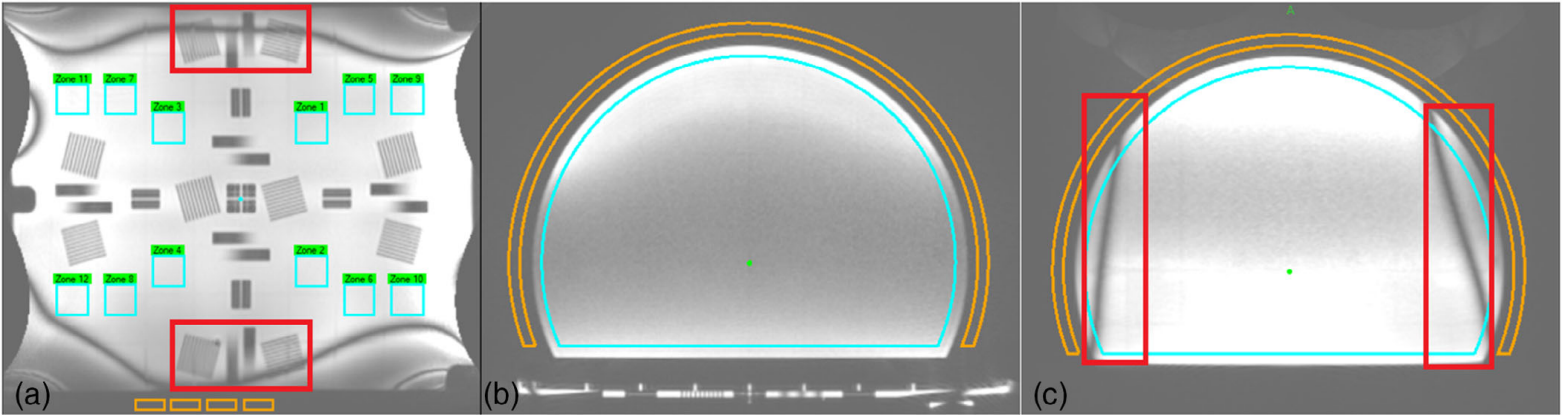
The uniformity analysis software interface. The orange and blue regions of interest (ROIs) measure the background noise and signal, respectively. The red areas indicated in (a) and (c) are banding artifacts. The base (a) of the phantom uses 12 small ROI regions. Both the axial (b) and sagittal (c) scans of the upright section use one large ROI.

The uniformity analysis depicted in Figure 2 is essential for MRI QA and is used to quantify the consistency of the magnetic field across the entire imaging volume. A region of interest (ROI) is selected outside the phantom to measure the standard deviation of the background noise, which is necessary for determining the signal‐to‐noise ratio (SNR). Other ROI(s) are then drawn by the software and are used to determine the maximum and minimum signal intensity. A smaller difference between these values indicates a greater uniformity value.

The sagittal and coronal banding artifacts in the TruFi sequence are a direct result of its extreme sensitivity to B0​ magnetic field inhomogeneities.^19^ The banding artifact creates an unwanted pattern (as shown in red areas in Figure 2a,c) that interferes with the phantom data. Since this false modulation (i.e., alternating dark and light pattern) is not related to the phantom structure, making the calculation unreliable, the best solution is to exclude the specific image zones that contain the artifact. Hence, in our coronal uniformity analysis, zones 9–12 (Figure 2a) were excluded due to the banding artifact that occasionally invades those ROIs.

#### 2.3.3 Slice thickness

**FIGURE 3:**
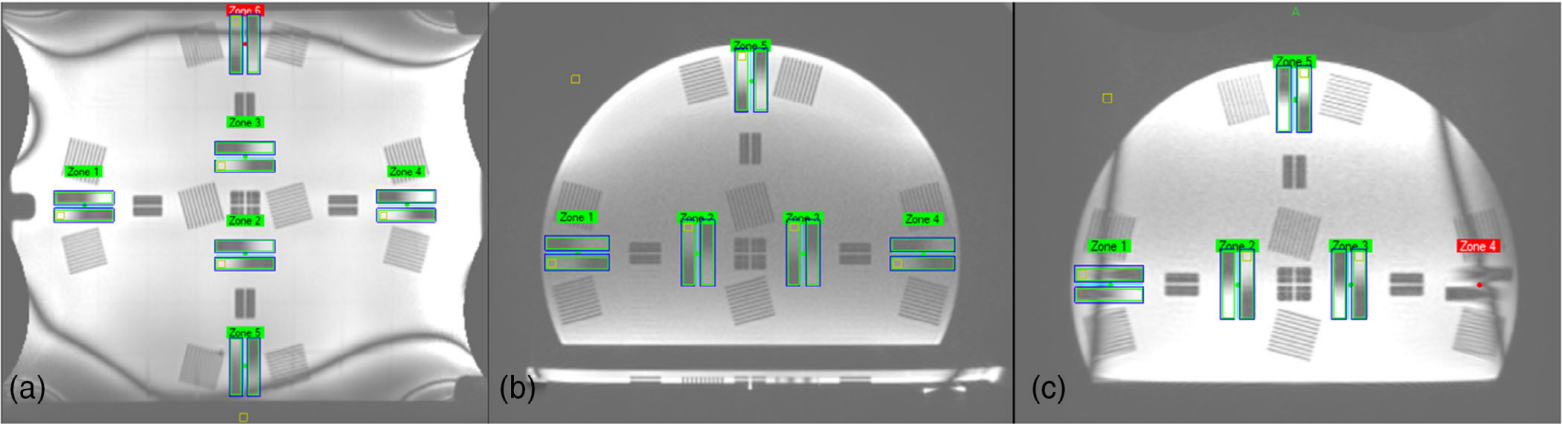
Slice thickness analysis in Modus QA software for the base (a) and upright (axial (b), sagittal (c)) sections.

The Insight Phantom includes five pairs of ramps for the slice thickness test (Figure 3). A line profile is created for each pair of ramps and the inflection points between the ramps’ different saturations are used to calculate the image thickness. Nominal slice thickness was 1.5 mm with daily tolerance of ±1 mm.

For each ramp in a pair, a rectangular ROI encompassing the ramp calculates the average pixel value in each row, and by extension the raw ramp profile can be obtained. The image slice only contains part of the ramp's gradient, and this is denoted as the “transition slope” in the ramp profile. On either side of this slope are two areas where the signal plateaus, and these are used as boundaries to determine the measured slice thickness. Then, we can calculate the percent difference between the measured slice thickness and the nominal slice thickness, the value used in the imaging sequence.

For coronal and sagittal scans, some zones may exhibit banding artifacts that can interfere with accurate slice thickness measurements. For the sagittal images, only zones 2, 3, and 5 (Figure 3c) were included due to imaging artifacts. Similarly, zones 5 and 6 (Figure 3a) were excluded in the coronal images.

For each daily scan, slice‐thickness values from all usable ramps were averaged to obtain a single daily slice‐thickness value. A zone‐wise assessment confirmed that the artifact‐free regions produced consistent measurements, with no systematic differences across spatial locations. Zones affected by banding or edge‐of‐FOV distortions were excluded from the averaged metric. As illustrated in Figure S1, the dashed horizontal line represents the daily mean slice thickness (calculated by averaging all artifact‐free zones for that scan), enabling visual comparison across zones. Zones 1–4, located centrally within the FOV, demonstrate consistent slice‐thickness values clustered closely around the daily mean. Zone‐wise analysis and statistical comparison against the central‐zone reference (two‐sided, *p* < 0.01) showed no statistically significant differences across usable zones, confirming spatial uniformity of slice thickness. In contrast, Zone 5 exhibited reduced measured thickness due to banding and edge‐of‐FOV artifacts and was therefore excluded from the daily QA aggregation. Overall, slice‐thickness values were spatially uniform across clinically usable zones, and zone‐to‐zone variability did not materially affect daily QA outcomes.

#### 2.3.4 Spatial resolution

Spatial resolution is another critical parameter in image quality assessment and is indicative of the ability to resolve small or closely spaced objects. The software uses a modulation transfer function (MTF) to evaluate the image spatial resolution. The spatial resolution is evaluated by analyzing groups of line pairs each within a 7.5 cm^2^ ROI.

**FIGURE 4:**
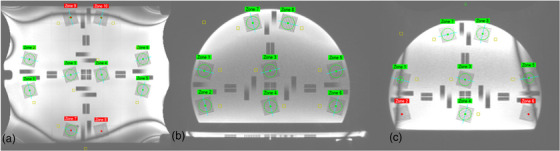
Spatial resolution analysis for the base phantom is shown in the coronal view (a), and for the upright phantom in the axial (b) and sagittal (c) views. The smaller orange regions of interest (ROIs) are used to calculate the *σ* and *S*.

Banding artifacts degrade image resolution and impact MTF (modulation transfer function) measurements by obscuring details. This is evident in Figure 4, particularly in Zones 7–10 of the coronal view and Zones 1, 2, 5, and 6 of the sagittal view. By ignoring certain zones, we can ensure that the MTF measured accurately reflects the true resolution capabilities of the system. Coronal scans were calculated using Zones 1–6 (Figure 4a) and sagittal scans using Zones 3, 4, 7, and 8 (Figure 4c).

A zone‐wise analysis demonstrated that spatial resolution was consistent across the central and mid‐phantom regions. The majority of zones exhibited MTF values within a narrow range (Figure S2), with no statistically significant differences compared with the central reference zones (Zones 1–6; *p* < 0.01). Peripheral zones, particularly those near the edge of the field of view (e.g., Zones 7 and 10), showed visibly reduced MTF values, consistent with expected decreases in SNR and gradient uniformity at the phantom boundary. These artifact‐affected zones were excluded from the daily averaged MTF metric.

#### 2.3.5 Geometric distortion

The Insight Phantom contains a grid of intersection points used to calculate the difference between the detected points and the known phantom coordinate. The grid itself is made up of squares with a side length of 5 cm. The software analyzes the distortion within concentric circular ROIs of radius 210, 310, and 410 mm. The base can be analyzed at a 510 mm radius due to its larger size.

**FIGURE 5:**
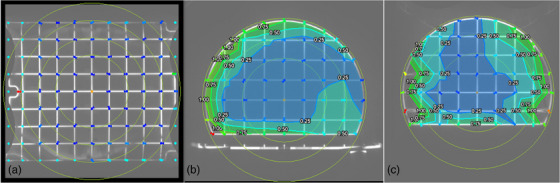
Distortion analysis for the base (a) and upright (axial (b), sagittal (c)) sections.

It has been noted that for any coronal scans, certain points at the edge of the FOV have been detected erroneously and should have been excluded (Figure 5a). These points do not contribute to an accurate distortion measurement.

For monthly QA, the passing criterion for distortion is 1 mm within 200 mm diameter and 2 mm within 350 mm diameter. Accordingly, for daily imaging QA, we analyzed the distortion within 210 mm, within 310 mm diameter, as well as the region in between 210 and 310 mm.

## 3 RESULTS

### 3.1 Alignment

Table 2 shows the mean and standard deviation (SD) of alignment and twist results for coronal, axial, and sagittal scans.

**TABLE 2:** Alignment results.

Scan (mean ± SD)	*x* (mm)	*y* (mm)	*z* (mm)	Twist (°)
Axial	0.61 ± 0.48	0.59 ± 0.62	−0.71 ± 0.42	−0.04 ± 0.08
Sagittal	0.27 ± 0.83	0.55 ± 0.87	−0.68 ± 0.43	0.06 ± 0.08
Coronal	0.54 ± 1.92	0.26 ± 1.77	−0.21 ± 1.52	0.00 ± 0.11

*Note*: The mean and standard deviation (SD) of alignment and twist results for the coronal, axial, and sagittal planes, respectively. Twists for coronal, axial, and sagittal scans are about the *y*, *z*, and *x* axis, respectively.

### 3.2 Uniformity

**FIGURE 6:**
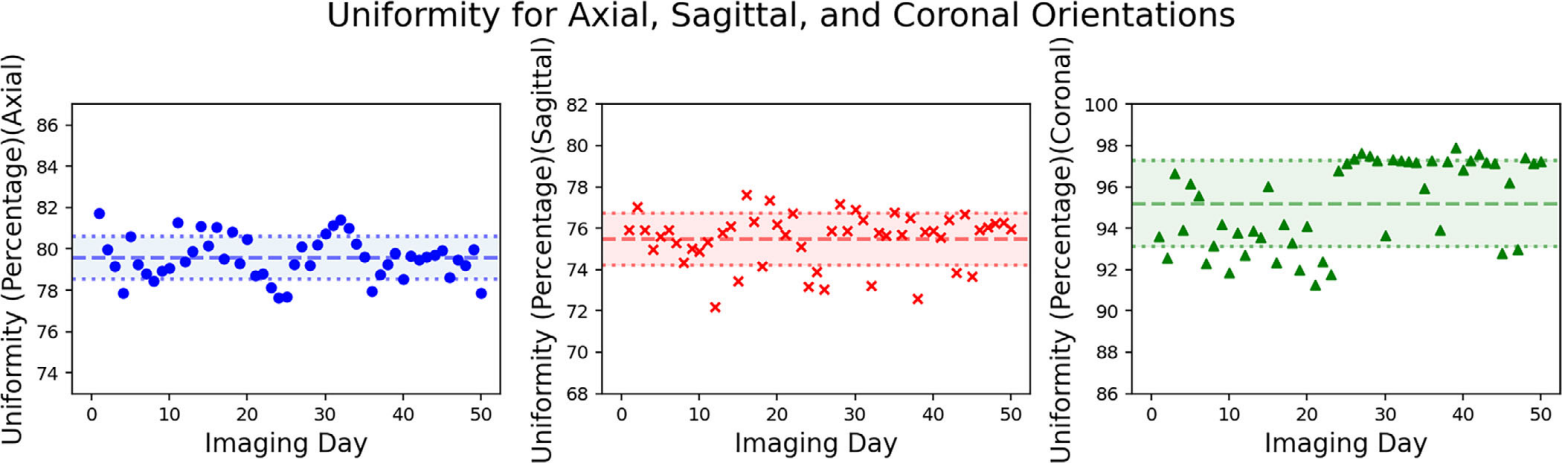
Uniformity results for axial, sagittal, and coronal orientations.

As shown in Figure 6, the uniformity values for axial, sagittal, and coronal orientations were 79.57 ± 1.02, 75.48 ± 1.25, and 95.19 ± 2.09, respectively.

### 3.3 Slice thickness

**FIGURE 7:**
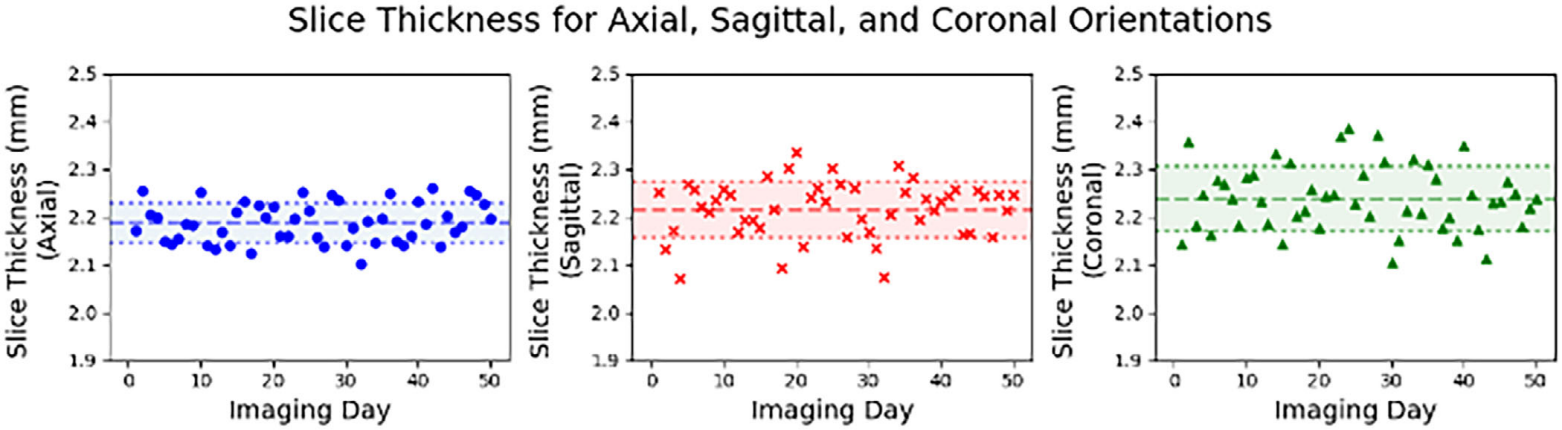
Slice thickness results for axial, sagittal, and coronal orientations.

Values for slice thickness were 2.19 ± 0.04, 2.22 ± 0.06, and 2.24 ± 0.07 mm for axial, sagittal, and coronal imaging orientations, respectively (Figure 7).

### 3.4 Spatial resolution

**FIGURE 8:**
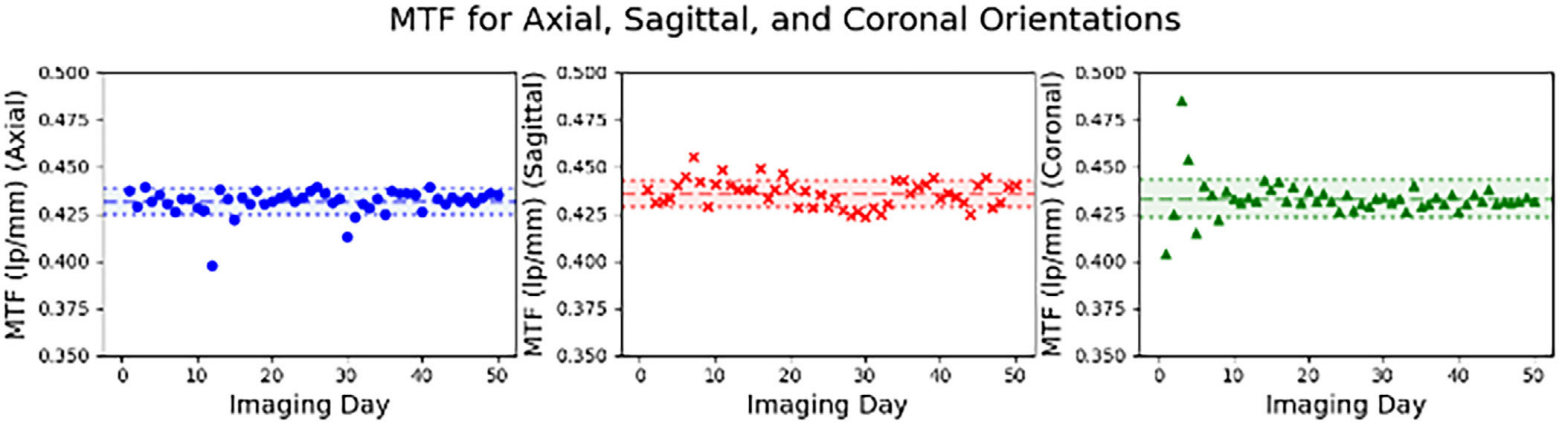
Modulation transfer function (MTF) values for axial, sagittal, and coronal orientations.

Figure 8 presents the MTF values for axial, sagittal, and coronal orientations that were 0.431 ± 0.007, 0.436 ± 0.007, and 0.433 ± 0.010 lp/mm, respectively.

### 3.5 Distortion

**FIGURE 9:**
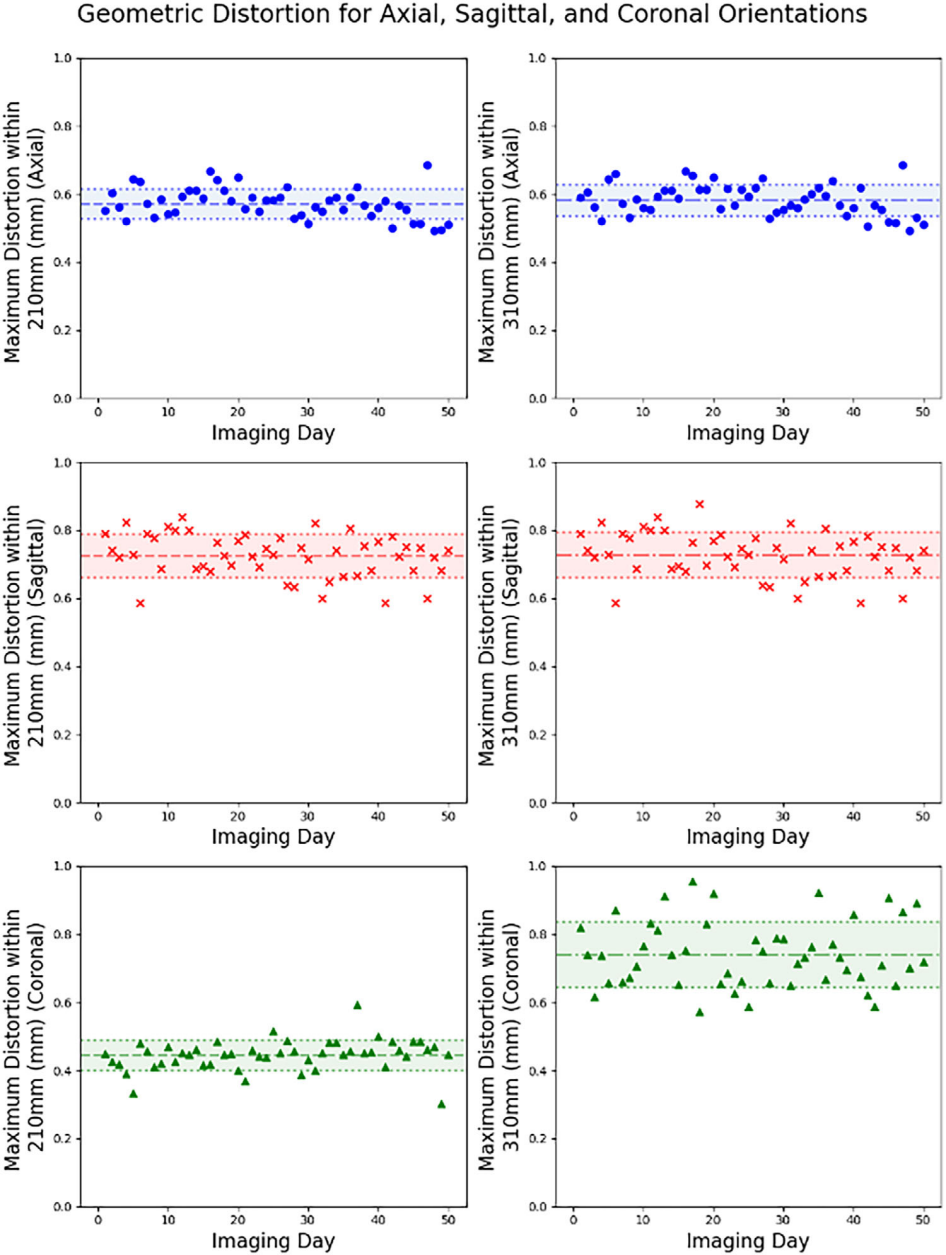
Maximum distortion values for axial, sagittal, and coronal orientations within 210 mm (left) and 310 mm (right).

Figure 9 shows the maximum distortion values for axial, sagittal, and coronal scans. Within a 210 mm diameter, the average maximum distortion value for axial, sagittal, and coronal orientations were 0.57 ± 0.04, 0.73 ± 0.06, and 0.45 ± 0.04 mm, respectively. Similarly, the average maximum distortion values within a 310 mm diameter are 0.58 ± 0.04, 0.73 ± 0.07, and 0.74 ± 0.10 mm. No maximum distortions within 210 or 310 mm were greater than or equal to 1 mm.

## 4 DISCUSSION

Within this study, over 3 months’ worth of data was collected and analyzed through the QUASAR MRgRT Phantom Insight software with set scan parameters. The inclusion of the coil bridge along with the phantom's flush design provides an easy and repeatable setup allowing therapists and physicists to utilize it reliably in daily QA. The physical size of the phantom also allows for QA to encompass a much larger FOV, which is useful for analyzing image uniformity and geometric distortion at locations farther away from the isocenter.

The phantom's alignment, as measured within the software, is in good agreement within acceptable range (mean deviations less than 1 mm). Any large sources of error in alignment are likely attributed to factors such as laser inaccuracies or misalignment during patient setup by therapists. The larger SD observed in the coronal orientation could mainly be due to uncertainty in detecting the center in the base plate. Another reason for misalignment could possibly be due to the phased array coils wrapping around the phantom, as pulling them too taught can cause them to bow and shift/rotate the phantom slightly. Values for twist are also very consistent and remain below ±0.1° for each imaging orientation.

Uniformity values of 79.57 ± 1.02 and 75.48 ± 1.25 were achieved for the axial and sagittal scans, respectively, and the coronal scans yielded a value of 95.19 ± 2.09. Uniformity is very dependent on imaging orientation and heavily influenced by how close the torso coils wrap the phantom. It is important to note that, despite being lower for certain scans, this uniformity value remains consistent over time, which supports the reliability of long‐term assessments. For coronal scans, uniformity results in some zones are occasionally affected by banding artifacts (Figure 2a), leading to false representations of signal loss or gain. The exclusion of certain uniformity zones is chosen to avoid interference with other image quality features and to ignore any banding artifacts. Adjustment of the positions of the outer zones can mitigate the impact of mentioned artifacts and provide a more accurate uniformity measurement.

Slice thickness results are around 50% greater than the nominal thickness (1.5 mm) on average, and this can be attributed to using the clinical 3D TruFi scan to perform the imaging daily QA. A more accurate slice thickness measurement using T1‐weighted spin echo 2D acquisition is done during our monthly imaging QA. The 3D TruFi scan was chosen for daily imaging QA mainly to check the consistency of the slice thickness rather than accuracy. The measured slice thicknesses of each imaging orientation are 2.19 ± 0.04, 2.22 ± 0.06, and 2.24 ± 0.07 mm for axial, sagittal, and coronal scans, respectively. With tightly clustered values for slice thickness measurements, the Insight Phantom proves to be a reliable tool to measure consistency, and future improvements in the software and scan parameters could allow for more accurate values.

Spatial resolution measurements are heavily impacted by artifacts and signal gradients as well, and therefore some zones have been excluded from the final measurements as mentioned in the Methods and Results sections. These values are also very tightly clustered at 0.431 ± 0.007, 0.436 ± 0.007, and 0.433 ± 0.010 lp/mm for axial, sagittal, and coronal scans, respectively. The TruFi imaging sequence has a shorter repetition time and a greater bandwidth, which decreases the signal amplitude and increases the noise. This decreases the SNR, resulting in a lower measured MTF.^20^

Geometric distortion measurements were well within the threshold values, that is, maximum values being less than 1 mm within 10 cm radius and 2 mm between 10 and 17.5 cm radius. The large FOV of the Insight Phantom particularly helps measure geometric distortion without having to image at multiple different phantom locations.^18^, ^19^, ^20^, ^21^, ^22^, ^23^, ^24^

The size of the Insight Phantom allows for a much more in‐depth analysis of imaging characteristics at the boundaries of the bore, and its design and accessories make setup and analysis efficient, easy, and timesaving. There are limitations to analyzing at such a large FOV, including the appearance of artifacts that makes valuable imaging zone data on coronal and sagittal scans unusable. Poor image quality and bad alignment can also cause the results to suffer dramatically, as even a very small twist will render images insufficient for analysis. Furthermore, the TruFi sequence used for daily imaging acquisition also result in lower‐than‐expected values for MTF and slice thickness values were higher than expected.

## 5 CONCLUSION

Our results demonstrate that the Insight Phantom offers a consistent, more comprehensive and efficient tool for daily MR‐Linac imaging QA. Within this work, we present the result of 3 months of imaging QA using the Insight Phantom to analyze the consistency, repeatability, and accuracy of imaging characteristic measurements. The size and shape of the phantom allow for multiple measurements to be taken in one short scan. Scanning with the TruFi 3D imaging sequence saves time, evaluates the clinical scan, and produces meaningfully consistent images that can be analyzed by Modus QA's software. The Insight Phantom acquires and analyzes imaging characteristics both consistently and in a timely manner, making it a practical and effective option for MRgRT daily QA.

Future extensions of this work will include longer‐term monitoring, multi‐institutional participation, and sensitivity analyses to common MR imaging issues such as coil element noise, off‐resonance effects, and spike artifacts.

## AUTHOR CONTRIBUTIONS

Borna Maraghechi contributed to the measurements, proofreading, and overall design and guidance of the work, as well as drafting the manuscript. Eli Grant contributed to programming, proofreading, and editing. Jiayi Liu contributed to data collection and editing. Ronak Etemadpour, Chengyu Shi, Huisi Ai, Percy Lee, Terence Williams, and An Liu contributed to proofreading and editing. All authors read and approved the final manuscript.

## CONFLICT OF INTEREST STATEMENT

The author declares no conflict of interest.

## Supporting information




**Figure S1**. Statistical analysis of slice‐thickness in coronal orientation. Each bar represents the measured slice‐thickness value obtained from an individual phantom zone.

**Figure S2**. Statistical analysis of spatial resolution (MTF) in coronal orientation. Each bar represents the measured resolution value obtained from an individual phantom zone.
