# Supplementary figures and images for: Evaluation of QUASAR Insight Phantom for daily imaging QA of MRgRT linacs
Source: J Appl Clin Med Phys. 2026 Feb 16;27(2):e70463. doi: 10.1002/acm2.70463 (PMC12910128; doi:10.1002/acm2.70463)

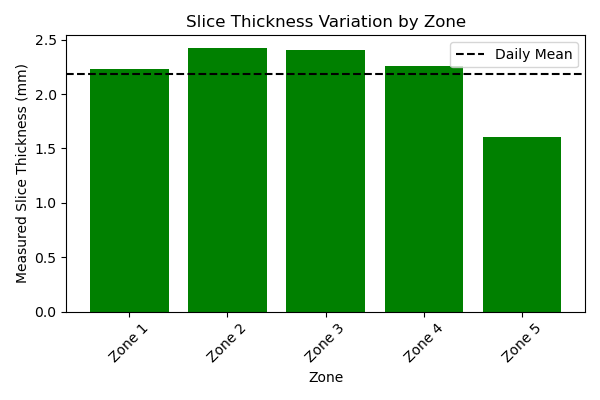

Supplement: Supplementary file 1 — Figure S1. Statistical analysis of slice‐thickness in coronal orientation. Each bar represents the measured slice‐thickness value obtained from an individual phantom zone. [file ACM2-27-e70463-s001.png]

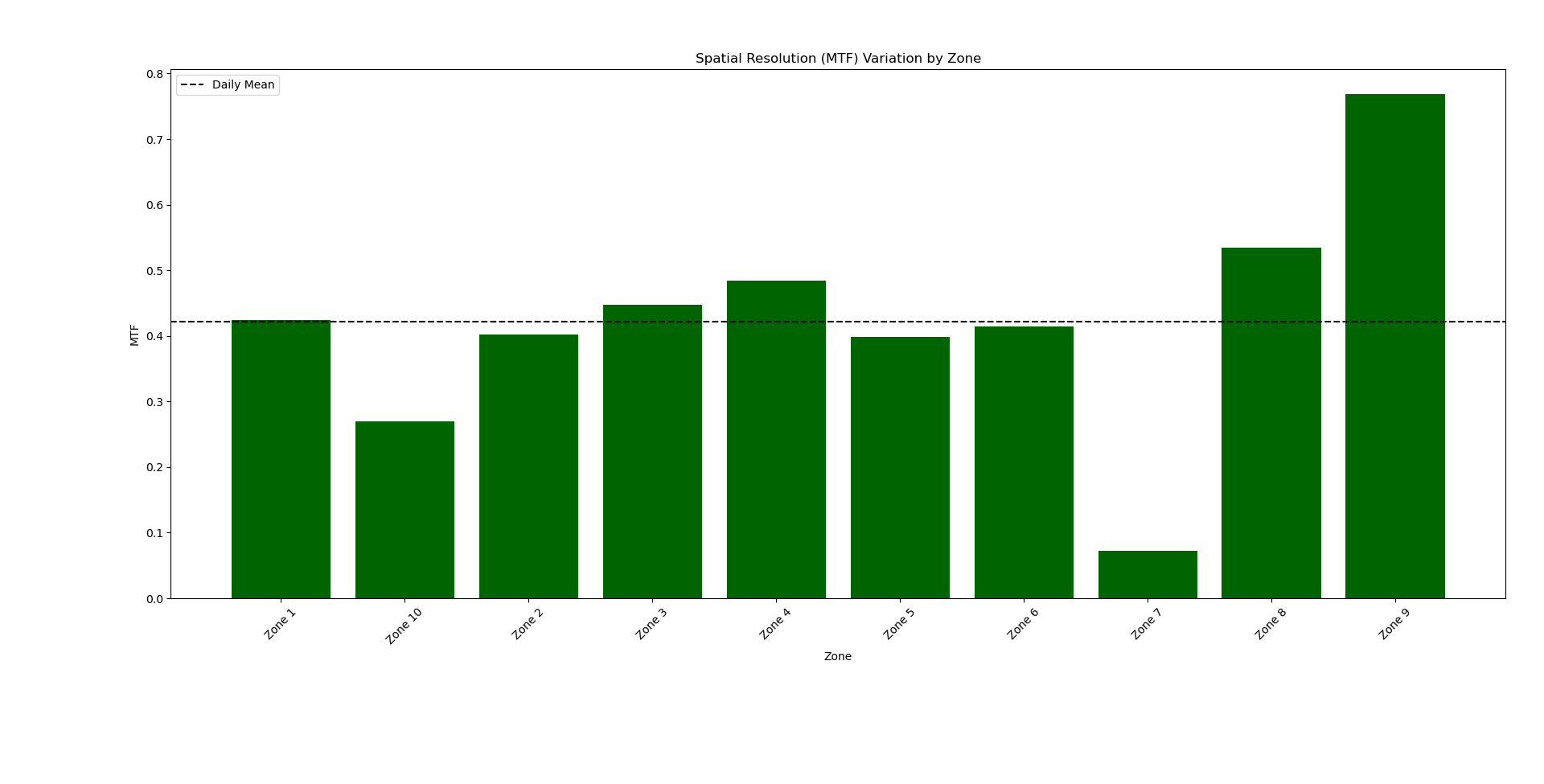

Supplement: Supplementary file 2 — Figure S2. Statistical analysis of spatial resolution (MTF) in coronal orientation. Each bar represents the measured resolution value obtained from an individual phantom zone. [file ACM2-27-e70463-s002.png]
